# Supplementary material for: Anterior cingulate cortex monitors action state and action content in complex associative learning
Source: eLife. 2026 Jul 10;14:RP105774. doi: 10.7554/eLife.105774 (PMC13354293; doi:10.7554/eLife.105774)
Supplement: Peer review history

# Editor report: eLife Assessment

**DOI:** 10.7554/eLife.105774.4.sa0

**Authors:** Alicia Izquierdo

**Affiliations:** University of California, Los Angeles United States

**Keywords:** Solid, Valuable

Huang and colleagues examined neural responses in mouse anterior cingulate cortex (ACC) during a discrimination-avoidance task. The authors present **valuable** findings that ACC neurons encode primarily "action content" over extended periods. The methodological approach is sound and the evidence in support of action state encoding is **solid**, though it is not conclusive to what extent ACC primarily encodes post-action events.

# Referee report: Reviewer #1 (Public review)

**DOI:** 10.7554/eLife.105774.4.sa1

Huang et al. examined ACC response during a novel discrimination-avoid task. The authors concluded that ACC neurons primarily encode post-action variables over extended periods, reflecting the animal's preceding actions rather than the outcomes or values of those actions. The authors have made considerable revision to address the raised the concerns. However, it appears that some important issues remain unresolved.

To what extent ACC neurons encode post action content remain as a major concern. This may be at least partially attributed by the analysis methods. If I understand it correctly, the authors compared pre- vs post-event neural activity and looked for significant changed. By default, this is to look for post-event changes, rather than pre-event. As a result, it would lead to the conclusion 'Our study also reveals that ACC neurons play a limited role in encoding pre-action variables associated with decision-making or planning, as evidenced by their minimal responses to auditory cues and the modest activity changes prior to shuttle initiation'.

To determine whether ACC encode pre-action variables or planning, different time windows should be used in the analysis.

# Referee report: Reviewer #2 (Public review)

**DOI:** 10.7554/eLife.105774.4.sa2

Summary:

Huang et al recorded anterior cingulate cortex activity in mice while they performed a shuttle escape task. The task utilized two auditory cues, each of which informed the mice to stay or escape depending on which side they were on, and incorrect responses were punished by shock administration. Analyses focused on ACC neurons that fired when mice crossed the shuttle box in either direction (A-->B or B-->A), coined "action state", or when mice crossed in one direction but not the other, coined "action content". The authors characterized these populations, and ACC firing changes mostly occurred around the time of shuttle crossing. This work will likely be of broad interest to those who are interested in neocortical neurophysiology broadly, anterior cingulate cortex specifically, and their contributions to learning about actions. The task is well-designed and provides a nice background for neurophysiological recordings. The authors leveraged these strengths in characterizing the neural populations that fire to shuttle crossings in both directions vs one direction.

Strengths:

The factorial design nicely controls for sensory coding and value coding, since the same stimulus can signal different actions and values.

The figures are well presented, labeled, and easy to read.

Additional analyses, such as the 2.5/7.5s windows and place-field analysis, are nice to see and indicate that the authors were careful in their neural analyses.

The n-trial + 1 analysis where ACC activity was higher on trials that preceded correct responses is a nice addition, since it shows that ACC activity predicts future behavior, well before it happens.

The authors identified ACC neurons that fire to shuttle crossings in one direction or to crossings in both directions. This is very clear in the spike rasters and population scaled color images. While other factors such as place fields, sensory input, and their integration can account for this activity, the authors discuss this and provide additional supplemental analyses.

# Referee report: Reviewer #3 (Public review)

**DOI:** 10.7554/eLife.105774.4.sa3

Summary:

The authors record from the ACC during a task in which animals must switch contexts to avoid shock as instructed by a cue. As expected, they find neurons that encode context, with some encoding of actions prior to the context, and encoding of neurons post-action. The primary novelty is dynamic encoding of action-outcome in a discrimination-avoidance domain, while this is traditionally done using operant methods.

Comments on revised version:

I appreciate subsequent responses to my comments and other reviewers. My comments are addressed, and at this point, I think readers can judge the work appropriately in context.

# Author comment: Author response

**DOI:** 10.7554/eLife.105774.4.sa4

**Authors:** Wenqiang Huang, Arron F Hall, Natalia Kawalec, Ashley Nicole Opalka, Jun Liu, Dong V Wang

**Affiliations:** Drexel University College of Medicine Philadelphia United States; Drexel University College of Medicine Philadelphia United States; Drexel University College of Medicine Philadelphia United States; Drexel University College of Medicine Philadelphia United States; Drexel University College of Medicine Philadelphia United States; Drexel University College of Medicine Philadelphia United States

The following is the authors’ response to the previous reviews

We thank the reviewers for their additional feedback. Below, we provide detailed responses to each reviewer’s major concerns. In addition, we identified an error in the previously submitted Fig. 6C and have corrected the X-axis labels accordingly.

**Public Reviews:**

**Reviewer #1 (Public review):**

Motion-related signal in ACC: the new Fig. 2E looks good, but it is hard to visualize how it is just a reordering of the old Fig. 5C.

We thank the reviewer for this feedback. Fig. 2E and the original Fig. 5C do bear resemblance. The primary difference is the temporal window and organization of the data. In the original Fig 5C, the time window was only ± 5 sec whereas Fig. 2E is ± 30 sec. The main objective we aim to highlight is that ACC shows both activation and inhibition in response to shuttle on an extremely prolonged order, up to 30 sec. Data is sorted to separate inhibition and activation to illustrate the sustained activity persists for both populations.

All categories in the new Fig. 4D appear to respond to shuttle initiation, with less than 1s latency. For example, type 2a/2b consists of 40% of the population and their response to movement onset is apparent. Thus, it is not clear whether most neurons respond to shuttle crossing as described in the manuscript.

We thank the reviewer for drawing attention to this discrepancy. It was not our intention to strike comparison between shuttle initiation versus shutting crossing responses across neurons, and we do not dispute that ACC responds to both events. While shuttle initiations and crossings provide a consistent temporal alignment point, they do not define the temporal focus of much of our analyses. Given that most shuttle responses terminate within ~2 sec, the extended windows analyzed (i.e. ± 5 sec; Fig. 4) largely reflect post-action ACC activity. Overall, although ACC neurons show mixed responses to initiations or crossings, the most consistent feature is prolonged modulation that persists beyond shuttle termination. We have revised the text to reflect this focus.

Given this and the reviewer’s feedback, we further examined whether ACC activity is more strongly aligned with shuttle initiation, crossing, or termination. To determine which shuttle event (initiation, crossing, or termination) captured the most acute changes in ACC neuronal firing, we conducted an event-locked modulation analysis (Fig. S4). Our results showed that shuttle crossing was associated with the largest fraction of significantly modulated ACC neurons (Fig. S4). These findings suggest that shuttle crossing represents the most prominent event for ACC engagement during shuttle behaviors.

Could the authors use relatively simple analysis, such as comparing spike rate before and after crossing, or before and after initiation, to quantify the response properties of each neuron? This could also help validate the classification analysis performed in Fig. 4.

As mentioned above, we have added a new supplemental figure to directly address this question (Fig. S4).

**Reviewer #2 (Public review):**

I think the authors did a very admirable job revising the manuscript. It is much improved. However, I believe a formal analysis of action-state versus action-content neurons on A-->B versus B-->A crossing is still warranted. I appreciate the fact that this analysis may not be as reliable with smaller ensemble sizes, but with careful pseudo-ensemble and resampling approaches, such an analysis would go a long way towards increasing the strength of evidence.

At present, we are not sure what the reviewer means as “formal analysis”. Below is our best effort in addressing this concern.

Firstly, in our first revised manuscript, we implemented a generalized linear model-based classification of action-content and action-state neurons using direction specific regressors. Specifically, this analysis classified neurons as action-content or action-state based on coefficient contrasts (Δβ), with appropriate statistical testing and multiple comparison correction (see Methods; Fig. 7 C–E). Neurons were classified as action-content neurons if the corrected p-value for Δβ was significant and the absolute effect size exceeded a predefined threshold (|Δ *β* |> 0.5). Neurons were classified as action-state neurons if Δβ was not significant but both β1 and β2 were individually significant after correction. We believe our generalized linear model-based classification offers a sophisticated and formal classification of these two neurons classes.

Subsequently, we performed an SVM decoder to distinguish A→B from B→A shuttles. Decoding accuracy depended on action-content neurons, as their removal drastically decreased decoding accuracy, whereas removal of non-action-content neurons had no effect, further strengthening the conclusion that these populations encode distinct information.

In the updated revision, we performed an additional SVM decoding analysis while controlling for unequal neuronal population sizes between action-state and action-content neurons (Fig. S8). Specifically, we constructed pseudo-ensembles by randomly resampling neurons within each category and training SVM decoders on size-matched ensembles. Decoder performance was evaluated across repeated resamples to generate distributions of accuracy. We found that only decoders using action-content neuronal activity predicted shuttle content with high accuracy (>95%), whereas decoders trained using non-action-content neurons performed at chance levels (Fig. S8).

**Reviewer #3 (Public review):**

The only remaining comment that was not addressed pertains to anatomy and recording details. Some electrodes appear to be clearly in M2 (Fig 2A), and the tetrodes were driven each day. I would strongly suggest that this be included as a further limitation, particularly given the statement on line 178.

We thank the reviewer for this feedback. In the previous revision, we added a supplemental figure showing tetrode locations for each mouse (Fig. S2) and described recording details in the Methods (Lines #481–488). We agree that this should also be noted as a limitation, and we have now added this to the Discussion (Lines #384–388).
